# The Clinical Effect of Digital Dorsal Fascial Island Flap Combined With Crossfinger Flap for Repairing Distal Degloving Injury and Sensory Reconstruction

**DOI:** 10.3389/fsurg.2021.732597

**Published:** 2022-01-17

**Authors:** Ruizheng Hao, Yongxin Huo, Hui Wang, Wei Liu

**Affiliations:** The Department of Hand Surgery, The Second Hospital of Tangshan, Tangshan, China

**Keywords:** distal degloving injury, digital dorsal fascial island flap, cross-finger flap, range-of-motion, visual analog scale score

## Abstract

**Background:**

To explore the clinical effect of digital dorsal fascial island flap combined with crossfinger flap to repair distal degloving injury and sensory reconstruction.

**Methods:**

A total of 19 patients with distal fingertip degloving injuries treated with digital dorsal fascial island flap combined with crossfinger flap in our hospital from April 2018 to August 2020 were retrospectively included. Semmes–Weinstein (SW) monofilament and static two-point discrimination (S-2PD) tests, active range-of-motion (ROM) of the fingers, cold intolerance, visual analog scale (VAS) score patient complications, and patient satisfaction were evaluated.

**Results:**

Five cases with post-operative flap blisters were treated at the time of dressing changes until successful scab formation. Three cases with post-operative arterial crisis of finger arterial dorsal branch vessel were relieved after suture removal and tension reduction. All other skin flaps and skin grafts survived. Nineteen patients received follow-up between 3 and 26 months (average 14.6 months). The active ROM of metacarpophalangeal (MCP) and interphalangeal (IP) joints of the injured fingers were satisfactory.

**Conclusion:**

The digital dorsal fascial island flap combined with the crossfinger flap for repairing the distal degloving injury of the distal segment of the finger is a good surgical method, which is simple and easy to operate, can repair a large area of soft tissue defect, and obtain a satisfactory effect.

## Introduction

Distal degloving injury is a common hand surgery injury with various repair methods, but improper treatment will seriously affect the post-operative appearance and function (1). Although revision amputation is simple, the length defect of the finger is compromised after operation. Although abdominal pedicled tubular flap can preserve the length of the finger, the finger is edematous and the sensory recovery is poor after surgery. Free toenail flap transplantation is a difficult operation with high risk, great damage to patients, and high requirements for surgeons (2–6).

At present, the digital dorsal fascial island flap has been widely used to repair the soft tissue defect of the dorsal or ventral finger (7–10). The crossfinger flap can repair the soft tissue defect of finger palmar and reconstruct the flap sensation, and it has the advantages of simple operation and low-operation risk (11, 12). Therefore, we designed and used digital dorsal fascial island flap combined with crossfinger flap to repair 19 cases of distal degloving injury of 19 fingers. The operation was safe and all the flaps survived with good clinical effect.

## Materials and Methods

### Patients

A total of 19 patients with distal fingertip degloving injuries treated in our hospital from April 2018 to August 2020 were retrospectively included.

Inclusion criteria were patients (1) who had degloving injuries of 2–5 finger pads and nail bed defects; (2) who had a strong desire to preserve the length of their fingers and can accept no nail plate growth; and (3) with a wound that could not be effectively covered by a single flap. Exclusion criteria were patients with (1) rheumatoid arthropathy, (2) diabetes, and (3) tuberculosis history.

There were 13 men and 6 women. Patients ranged in age from 19 to 60 years (average age 36.2 years), with an average of 3.5 h from injury to surgery. The defects involved soft tissue detachment of 1/2 distal finger (*n* = 5), 2/3 of distal finger (*n* = 11), and 3/4 distal finger (*n* = 3). The defect size of distal fingertip degloving injury was in length from 2.3 to 4.6 cm and in width from 1.2 to 1.9 cm, with mild to severe contamination, nail bed damage, exposed tendons and phalanges, and no rupture of flexor and extensor tendon. Among them, six cases were combined with phalanx distal fractures (Table 1). The study was approved by the Ethics Committee of Tangshan Second Hospital (TSEY-LL-2020016). Signed informed consent was obtained from each patient.

**Table 1:** Demographic and surgical details of the patients.

**Case**	**Sex**	**Age**	**Type of injury**	**Injury finger (left/right)**	**Defect size (cm** **× cm)**		**Flap size (cm** **× cm)**		**Follow-up time (months)**
					**Palmar of finger**	**Dorsal of finger**	**Palmar of finger**	**Dorsal of finger**	
1	Male	24	Avulsion	Right index finger	2.0 cm × 1.8 cm	1.8 cm × 1.8 cm	2.2 cm × 2.0 cm	2.0 cm × 2.0 cm	15
2	Male	35	Crush	Left index finger	2.1 cm × 1.9 cm	1.9 cm × 1.9 cm	2.4 cm × 2.1 cm	2.1 cm × 2.1 cm	11
3	Male	19	Crush	Right ring finger	2.3 cm × 1.8 cm	2.0 cm × 1.8 cm	2.6 cm × 2.0 cm	2.2 cm × 2.0 cm	12
4	Female	21	Avulsion	Left middle finger	1.3 cm × 1.5 cm	1.4 cm × 1.5 cm	1.5 cm × 1.7 cm	1.5 cm × 1.7 cm	15
5	Male	26	Crush	Right ring finger	1.9 cm × 1.7 cm	1.8 cm × 1.7 cm	2.1 cm × 1.9 cm	2.0 cm × 1.9 cm	3
6	Female	26	Crush	Left index finger	1.6 cm × 1.6 cm	1.5 cm × 1.6 cm	1.8 cm × 1.8 cm	1.7 cm × 1.8 cm	18
7	Female	60	Avulsion	Left ring finger	1.7 cm × 1.8 cm	1.6 cm × 1.8 cm	1.9 cm × 2.0 cm	1.8 cm × 2.0 cm	12
8	Male	47	Crush	Right middle finger	2.2 cm × 1.9 cm	2.0 cm × 1.9 cm	2.5 cm × 2.1 cm	2.2 cm × 2.1 cm	8
9	Male	22	Avulsion	Right little finger	1.5 cm × 1.4 cm	1.3 cm × 1.4 cm	1.7 cm × 1.6 cm	1.4 cm × 1.5 cm	12
10	Male	25	Crush	Left index finger	1.2 cm × 1.7 cm	1.1 cm × 1.7 cm	1.3 cm × 1.9 cm	1.2 cm × 1.9 cm	26
11	Female	31	Avulsion	Right index finger	1.7 cm × 1.6 cm	1.5 cm × 1.6 cm	1.9 cm × 1.8 cm	1.7 cm × 1.8 cm	18
12	Male	52	Avulsion	Right middle finger	2.4 cm × 1.8 cm	2.2 cm × 1.8 cm	2.7 cm × 2.0 cm	2.4 cm × 2.0 cm	12
13	Male	21	Crush	Right index finger	1.5 cm × 1.6 cm	1.6 cm × 1.6 cm	1.7 cm × 1.8 cm	1.8 cm × 1.8 cm	16
14	Female	34	Avulsion	Right middle finger	2.0 cm × 1.7 cm	1.8 cm × 1.7 cm	2.2 cm × 1.9 cm	2.0 cm × 1.9 cm	13
15	Male	32	Crush	Left ring finger	1.5 cm × 1.7 cm	1.8 cm × 1.7 cm	1.7 cm × 1.9 cm	2.0 cm × 1.9 cm	15
16	Male	51	Avulsion	Right ring finger	1.2 cm × 1.6 cm	1.1 cm × 1.6 cm	1.3 cm × 1.8 cm	1.2 cm × 1.8 cm	18
17	Female	55	Crush	Right little finger	1.2 cm × 1.2 cm	1.1 cm × 1.2 cm	1.3 cm × 1.3 cm	1.2 cm × 1.3 cm	15
18	Male	48	Avulsion	Left index finger	1.8 cm × 1.5 cm	1.6 cm × 1.5 cm	2.0 cm × 1.7 cm	1.8 cm × 1.7 cm	18
19	Male	59	Crush	Right ring finger	2.1 cm × 1.9 cm	1.9 cm × 1.9 cm	2.3 cm × 2.1 cm	2.1 cm × 2.1 cm	20
Mean		36.2							14.6

### Surgical Methods

#### Flap Design

The digital dorsal fascial island flap contains the dorsal branch of the proper digital artery of the finger middle segment, and the flap was turned to cover the wound on the dorsal side of the distal finger. The dorsal branch of the proper digital nerve carried by the crossfinger flap was accurately anastomosed with the stump of the proper digital nerve in the recipient area (12), which was used to repair the palmar wound of injured finger. A two-leaf tile flap composed of the digital dorsal fascial island flap of the injured finger and the crossfinger flap was used to repair the degloving injury of the distal segment of the finger.

The surgery was performed by the same senior surgeon. Patients were administered brachial plexus block anesthesia. A tourniquet was applied to the injury-bearing arm. Wound debridement was performed to remove necrotic and contaminated tissue, nail matrix, and residual nail bed. A 1 mm Kirschner wire was used for fixation in cases of distal fractures (13).

**Figure 1:**
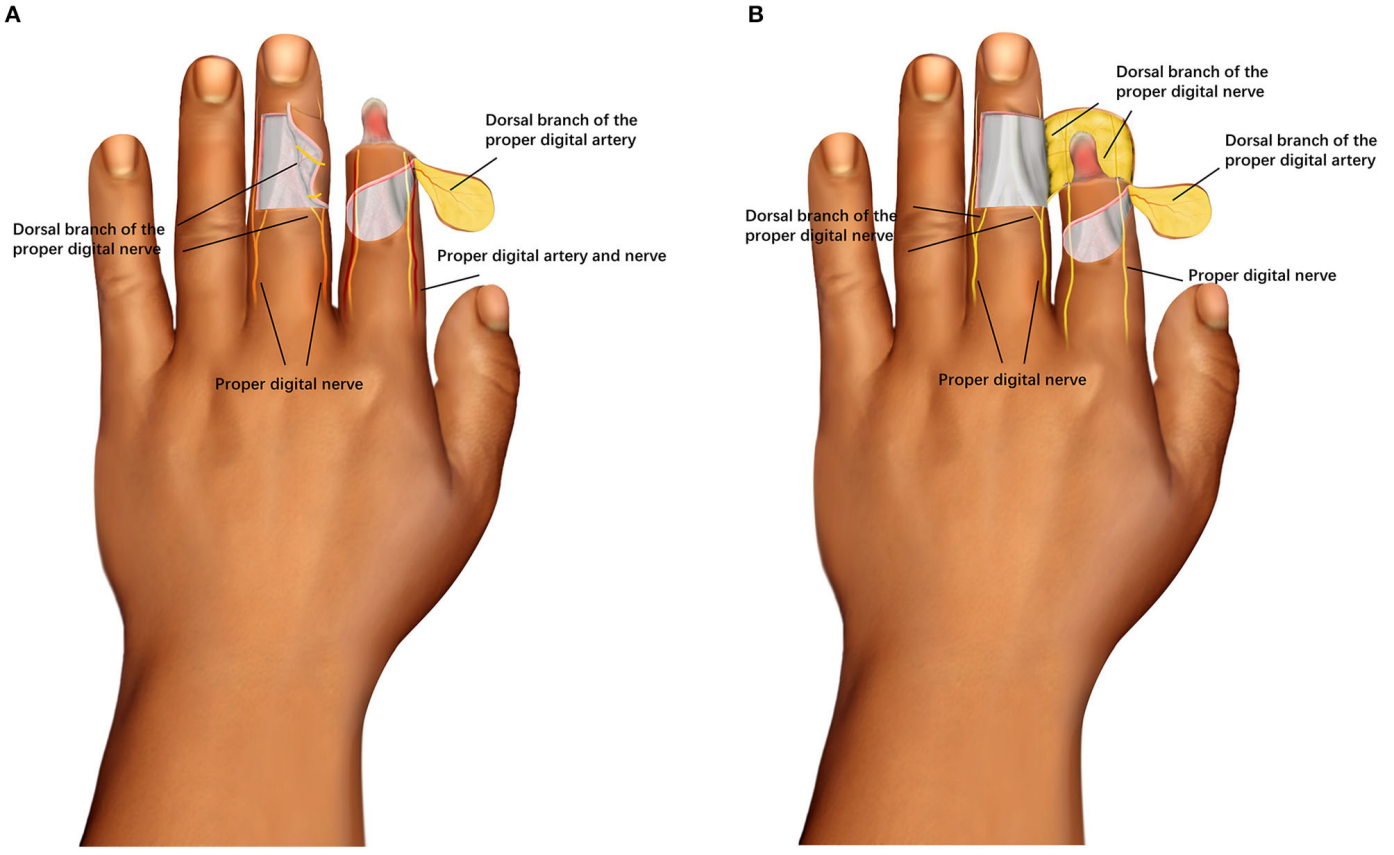
**(A)** According to the size of the palmar wound of the distal segment of the injured finger, a crossfinger flap is designed on the dorsal area of the adjacent middle segment of the finger. The pedicle contained the dorsal branch of the proper digital artery of the finger middle segment, and the flap was turned over to cover the palmar wound and finger tip of the distal segment of the finger. The digital dorsal fascial island flap was designed on the dorsal area of the same middle segment of the finger according to the size of the soft tissue defect on the distal dorsal side of the injured finger. The digital dorsal fascial island flap contains the dorsal branch of the proper digital artery of the finger middle segment, and the flap was turned to cover the wound on the dorsal side of the distal finger. **(B)** The crossfinger flap was turned over to cover the palmar wound and finger tip of the distal segment of the finger. The dorsal branch of the proper digital nerve carried by the crossfinger flap was accurately anastomosed with the stump of the proper digital nerve in the recipient area. The digital dorsal fascial island flap contains the dorsal branch of the proper digital artery of the finger middle segment, and the flap was turned to cover the wound on the dorsal side of the distal finger.

The digital dorsal fascial island flap was designed on the dorsal area of the same middle or proximal segment of the finger according to the size of the soft tissue defect on the distal dorsal side of the injured finger. The area of the flap was 10% larger than the dorsal surface of the finger. The rotation point was located on the lateral side of the transverse palmar striations of the distal interphalangeal (DIP) joint. The vascular pedicle was the dorsal branch of the digital artery and its surrounding fascia was about 0.5 cm in width (14, 15), and the pedicle was 0.8–1.0 cm in length. The opposite side of the pedicle should not exceed the midline of the contralateral side. The axis was the junction of the middle and outer half of the dorsal finger, which was parallel to the longitudinal axis of the finger. The skin and subcutaneous tissue were cut according to the design incision, and the superficial layer of the aponeurosis of the extensor tendon was sharply separated to the vascular pedicle of the digital artery dorsal branch flap, and the flap was turned over to cover the wound of the distal dorsal finger (Figure 1A).

According to the size of the palmar wound of the distal segment of the injured finger, a crossfinger flap was designed on the dorsal area of the middle segment of the adjacent finger. The area of the flap was about 12% larger than that of the palmar segment of the finger, and the pedicle of the flap was designed with a narrow pedicle of 0.8 cm. The pedicle contained the dorsal branch of the proper digital artery of the finger middle segment, and the flap was turned over to cover the palmar wound and finger tip of the distal segment of the finger (Figure 1B) (16). The dorsal branch of the proper digital nerve carried by the crossfinger flap was exactly consistent with the stump of the proper digital nerve in the recipient area. A two-leaf tile flap composed of the digital dorsal fascial island flap of the injured finger and the crossfinger flap was used to repair the detachment of the distal segment of the finger. The donor site of the flap was taken from the forearm full-thickness skin graft and packaged.

Post-operative infection prevention, swelling reduction, and symptomatic treatment were performed. The sutures were removed after 14 days, and the pedicle of the crossfinger flap was divided at post-operative 3 weeks, and functional exercises were performed.

### Evaluation of Outcomes

All tests were performed by the same senior surgeon. Sensibilities of the flap were measured using Semmes–Weinstein (SW) monofilament and static two-point discrimination (S-2PD) tests. The range-of-motion (ROM) of the fingers was measured with a standard hand goniometer, and the degree of flexion of the metacarpophalangeal and interphalangeal (IP) joints of the finger minus the degree of extension loss was compared with the other hand. Cold intolerance of the reconstructed finger was measured using the self-administered Cold Intolerance Severity Score (CISS) questionnaire (13, 17). The scores are grouped into four degrees (mild, moderate, severe, and extremely severe) corresponding to four ranges (0–25, 26–50, 51–75, and 76–100), respectively. Pain was assessed by visual analog scale (VAS). The appearance of the reconstructed finger and the donor site were assessed using the Michigan Hand Outcomes Questionnaire (18).

### Statistical Analysis

All analyses were performed using SPSS 23.0 software (SPSS, Inc., Chicago, IL). Paired *t*-test was used to compare the difference in the active ROM of MCP and IP joints of the injured fingers with those of the contralateral side. Two-tailed probability value of *p* < 0.05 was considered as statistically significant.

## Results

A total of 5 cases with post-operative flap blisters were treated at the time of dressing changes up to successful scab formation. Three cases with post-operative arterial crisis of finger arterial dorsal branch vessel were relieved after suture removal and tension reduction. All other skin flaps and skin grafts survived. Nineteen patients received follow-up between 3 and 26 months (average 14.6 months).

As shown in Table 2, the S-2PD score of dorsal finger flap was 6–11 mm (average 8.63 mm). The S-2PD score of the palmar finger flap was 5–10 mm (average 7.58 mm). The SWM score of the dorsal flap of the injured finger was 2.65–5.32 mm (average 4.11 mm). The SWM score of the palmar flap of injured finger was 3.23 to 5.13 mm (average 3.89 mm). Based on the CISS score, 17 patients with the dorsal flap of the injured finger reported no cold intolerance and 2 reported mild cold intolerance. A total of 15 patients of the palmar flap of injured finger reported no cold intolerance and 4 reported mild cold intolerance. According to the VAS score, 16 patients had no pain and 3 reported mild pain on the dorsal flap of injured finger. A total of 16 patients had no pain, 2 reported mild pain, and 1 experienced moderate pain on the palmar flap of the injured finger.

**Table 2:** Post-operative assessment of injured finger.

	**Palmar of finger**				**Dorsal of finger**			
**Case**	**S-2PD, mm**	**SWM**	**Cold intolerance**	**Pain**	**S-2PD, mm**	**SWM**	**Cold intolerance**	**Pain**
1	7	3.86	0	0	9	4.63	0	0
2	6	4.08	0	0	10	4.38	0	0
3	8	3.62	0	0	8	3.53	20	0
4	7	3.35	0	0	9	4.65	0	0
5	7	3.95	20	1	10	2.65	0	2
6	8	4.52	0	2	7	4.83	0	0
7	8	4.15	0	0	9	3.58	0	1
8	9	3.75	10	0	11	4.63	0	0
9	9	4.23	0	0	8	3.68	0	0
10	6	3.61	0	0	9	3.84	0	0
11	5	3.47	20	4	7	3.21	0	0
12	6	4.34	0	0	9	4.75	0	0
13	7	3.56	0	0	10	3.65	10	0
14	9	5.13	0	0	7	4.25	0	2
15	10	3.24	0	0	9	3.58	0	0
16	9	3.58	0	0	10	5.32	0	0
17	6	3.85	10	0	8	4.85	0	0
18	8	4.45	0	0	6	4.76	0	0
19	9	3.23	0	0	8	3.28	0	0
Mean	7.58	3.89			8.63	4.11		

*S-2PD, static 2-point discrimination; SWM, Semmes–Weinstein monofilament*.

The active ROM of metacarpophalangeal (MCP) and IP joints of the injured fingers were satisfactory (Table 3). No statistical differences were observed in the ROM of MCP, proximal interphalangeal joint (PIP), and DIP compared with that of the contralateral side (pMCP = 0.157, pPIP = 0.120, pDIP = 0.301). The quality of the activity of the injured fingers showed no abnormality.

**Table 3:** ROM assessment of the fingers.

	**ROM (degree)**					
**CASE**	**MCPI**	**MCPC**	**PIPI**	**PIPC**	**DIPI**	**DIPC**
1	87	88	105	107	70	70
2	86	87	108	108	78	79
3	88	90	108	107	72	68
4	87	89	106	108	66	69
5	87	85	108	108	68	68
6	89	90	103	100	72	69
7	88	88	96	105	60	67
8	89	87	102	104	58	65
9	89	89	102	103	75	68
10	89	88	105	105	68	68
11	86	89	106	106	69	70
12	90	88	105	107	62	68
13	87	87	98	102	69	71
14	85	86	106	108	68	72
15	86	89	105	106	68	69
16	85	87	103	100	72	70
17	86	88	100	98	73	71
18	90	92	105	106	68	68
19	89	87	102	104	69	72
Mean	87.53	88.11	103.84	104.84	68.68	69.58
t	1.476		1.635		1.064	
P	0.157		0.120		0.301	

*ROM, range of motion; MCPI, metacarpophalangeal joint of injury side; MCPC, metacarpophalangeal joint of contralateral side; IPI, interphalangeal joint of injury side; IPC, interphalangeal joints of contralateral side*.

According to the Michigan Hand Outcomes questionnaire, 15 patients were strongly satisfied (score of 5) and 4 patients were satisfied (score of 4) with the appearance, whereas 16 patients were strongly satisfied (score of 5) and 3 patients were satisfied (score 4) with the function of the reconstructed finger. There was no obvious complication except for the low sensitivity of the skin flap and no nail plate growth.

## Case Reports

### Case 2

**Figure 2:**
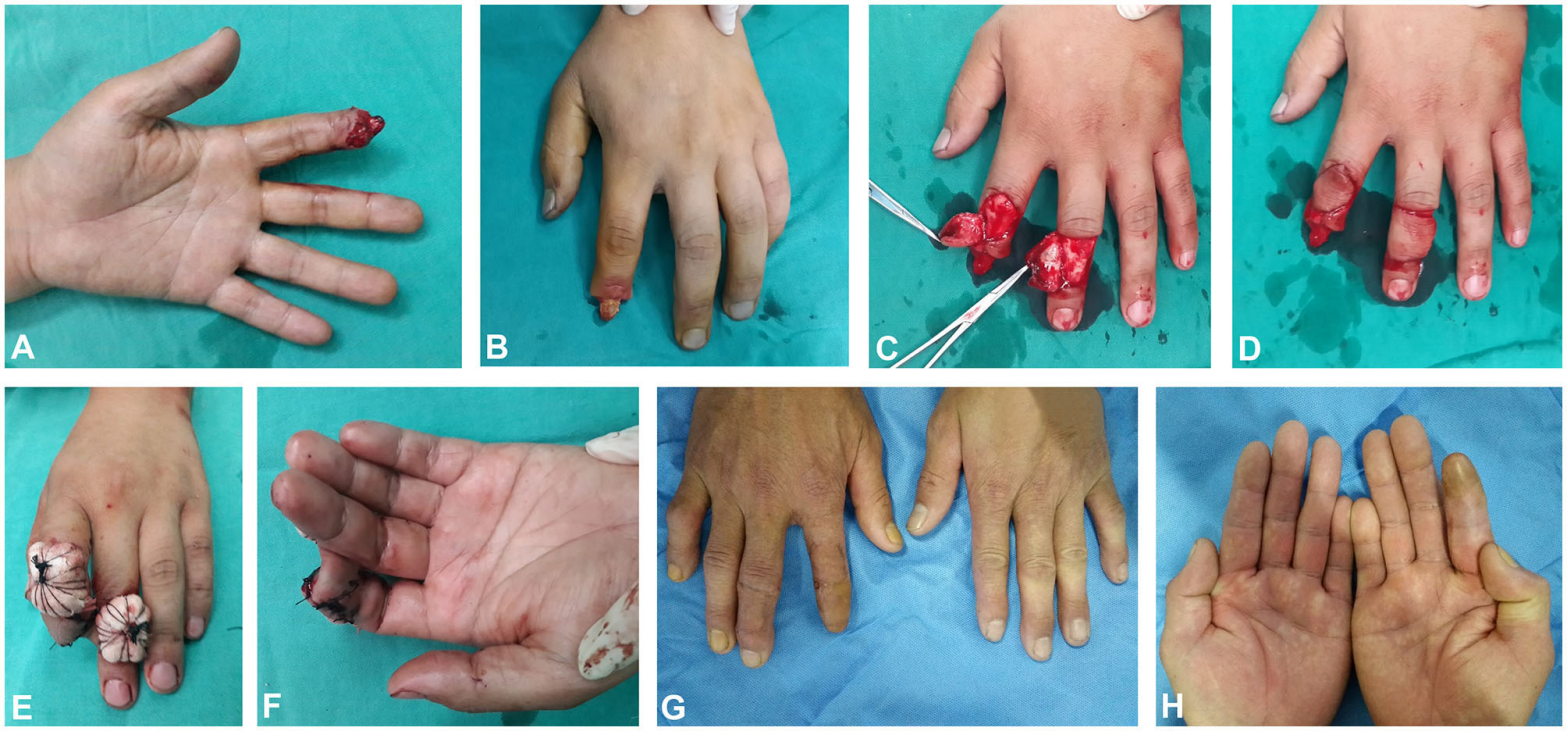
Flap of case 2. **(A,B)** The size of the defect were 2.1 cm × 1.9 cm on the palmar side and 1.9 cm × 1.9 cm on the dorsal side. **(C,D)** Skin flap cutting. **(E,F)** Skin flap suturing. **(G,H)** The appearance of the flaps 11 months later.

The subject was a 35-year-old male with avulsion of distal tissue of left index finger. The size of the defect was 2.1 cm × 1.9 cm on the palmar side and 1.9 cm × 1.9 cm on the dorsal side (Figures 2A,B). A 2.1 cm × 2.1 cm size digital dorsal fascial island flap of left ring finger was cut to cover the dorsal wound of the index finger and a 2.4 cm × 2.1 cm size crossfinger flap was cut to cover the index finger palmar wound (Figures 2C–F). The 11-month follow-up evaluation showed the fingers were plump and soft in shape. The S-2PD score of the dorsal and palmar flap of injured finger was 10 and 6 mm, respectively. The SWM score of the dorsal and palmar flap of the injured finger was 4.38 and 4.08 mm, respectively. The ROM of MCP, PIP, and DIP was 86, 108, and 78 degrees, respectively. Good appearance of fingers were observed (Figures 2G,H).

### Case 3

**Figure 3:**
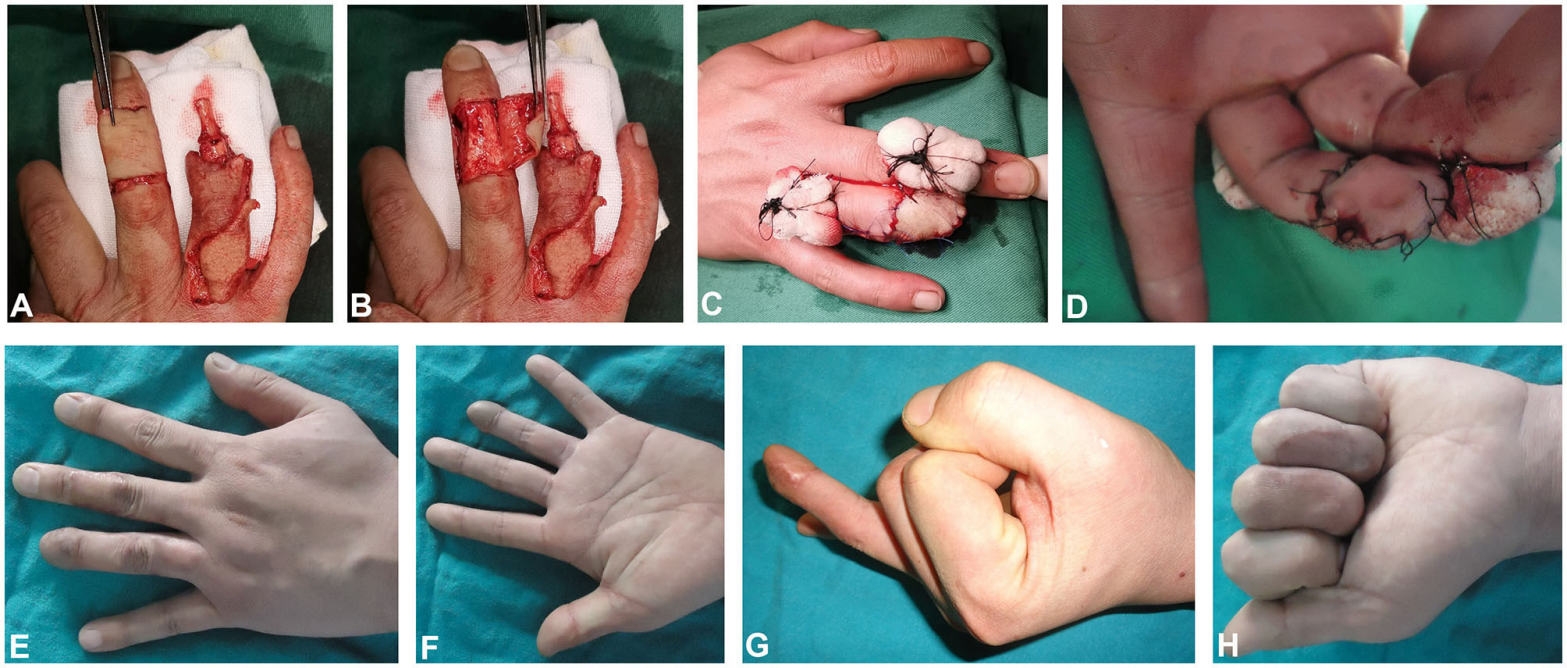
Flap of case 3. **(A,B)** Skin flap cutting. **(C,D)** Skin flap suturing. **(E–G)** The appearance of the flaps 6 months later. **(H)** The post-operative function 12 months later.

The subject was 19-year-old male with right ring finger crush injury. The size of the defect was 2.3 cm × 1.8 cm on the palmar side and 2.0 cm × 1.8 cm on the dorsal side. A 2.2 cm × 2.0 cm size digital dorsal fascial island flap of the left ring finger was cut to cover the dorsal wound of the ring finger and a 2.6 cm × 2.0 cm size crossfinger flap was cut to cover the ring finger palmar wound (Figures 3A–D). The 12-month follow-up evaluation showed the S-2PD score of the dorsal and palmar flap of injured finger was 8 and 8 mm, respectively. The SWM score of the dorsal and palmar flap of injured finger was 3.53 and 3.62 mm, respectively. The ROM of MCP, PIP, and DIP was 88, 108, and 72 degrees, respectively. Good appearance and function of fingers were observed (Figures 3E–H).

## Discussion

The commonly used V-Y advancement flap, crossfinger flap, fascial island flap, and abdominal flap have their own advantages, disadvantages, and indications (19). V-Y advancement flap, cross-finger flap, and fascial island flap are effective in repairing small soft tissue defects in the fingers; however, it cannot achieve a good treatment effect for a large area of degloving injury (20). Although abdominal flaps can repair large areas of skin defects, their appearance is edematous, and the skin texture is very different from finger skin. In most cases, the sensation cannot be reconstructed, and a second operation is often required to repair the flap in the later period (19). However, local cutaneous flap provides better color, texture, minimal tissue contraction, and a higher survival rate, and is appropriate in case of tendon or bone exposure (21).

The digital dorsal fascial island flap and the crossfinger flap are commonly used in hand surgery with reliable blood supply. The crossfinger flap anastomosed with dorsal digital nerve can make the finger more sensitive and restore the sensory function earlier (12). We combined the two flaps to repair the soft tissue defect degloving injury of the distal segment of the finger, which can repair the wound defect at one time and preserve the function of the finger to the greatest extent (22, 23), while reducing the damage to the donor site and recovering the function of the affected finger as soon as possible. This method can change the large damage into several small damage areas, and change the large defect area into two small defects from the three-dimensional point of view. This operation method is suitable for clinical application and has a high application value.

The advantages of this method were as follows. (1) The digital dorsal fascial island flap and the cross-finger flap pedicle include the dorsal branch of the proper digital artery of the middle finger segment and surrounding fascial tissues, with rich blood supply, reliable blood circulation, and high survival rate. (2) The flap is easy to dissect without damaging the main nerve and vessels (15, 24). (3) The skin flap was removed from the tissue around the adjacent wound. The skin color and texture were similar to that of the original finger wound surface. The affected finger had good appearance without edema after repair. (4) Crossfinger flaps can carry bilateral dorsal branches of the proper digital nerves and anastomose the proper digital nerves in the recipient area to reconstruct the protective sensation of the flaps. (5) The ROM of the DIP of the injured finger after surgery is not different from the contralateral finger. The finger preserves the length of the finger and also obtains good function. There are some disadvantages. (1) Fingertip onychostroma needs to be completely excised, and there is no growth of the nail plate after the operation, which affects the appearance. (2) The dorsal finger skin is thin, and the digital dorsal fascial island flap contains the dorsal branch of the proper digital artery. The vascular pedicle of the skin flap leads to slightly edematous appearance on one side of the finger in some cases. (3). A second operation is needed to divide the pedicle of crossfinger flap. (4). After crossfinger flap surgery, the injured finger cannot move due to the connection between the adjacent finger and the injured finger. Three weeks after the pedicles were cut, finger stiffness may occur and active functional exercises are required.

The precautions for the method in our study were as follows. (1) When dealing with the venous return disorder of the flap, the suture should be removed and the tension should be reduced in 1–2 days after the operation, and the pedicle should be opened without suture. (2) During the sensory reconstruction of the crossfinger flap, the dorsal branch of the proper digital nerve needs to be dissected for a certain length to achieve an anastomosis with the stump of the proper digital nerve in the recipient area (25, 26). The nerve separation and anastomosis should be performed under a microscope. (3) Remaining damaged nail beds should be removed during the operation to avoid the formation of ingrown nails later. (4) The skin graft packing pressure should be appropriate in order to avoid the pedicle pressure caused by force transmission, affecting the blood supply of the flap. (5). When suturing the two-leaf tile flap, do not sew under tension, cover the pedicle with oily gauze, and do not press it when bandaging. (6). The patients started active ROM exercises with the help of a physical therapist after 3 weeks. Tactile stimulation was applied to the recipient site and continued until the patient returned to work.

There were some limitations in our study. The sample size was small and there was no control group. The follow-up time of this study was 3–26 months with an average of 14.6 months, and some patients had a shorter follow-up time. Therefore, large randomized controlled trials remain to be done in the future.

In conclusion, the digital dorsal fascial island flap combined with the cross-finger flap for repairing the distal degloving injury of the distal segment of the finger is a good surgical method, which is simple and easy to operate, can repair a large area of soft tissue defect, and obtain a satisfactory effect.

## Data Availability Statement

The original contributions presented in the study are included in the article/supplementary material, further inquiries can be directed to the corresponding author/s.

## Ethics Statement

The study was approved by the Ethics Committee of Tangshan Second Hospital (TSEY-LL-2020016). The patients/participants provided their written informed consent to participate in this study. Written informed consent was obtained from the individual(s) for the publication of any potentially identifiable images or data included in this article.

## Author Contributions

RH and YH conceived of the study and contributed to manuscript writing. RH, YH, and HW participated in data collection. RH, YH, HW, and WL contributed to results interpretation. All authors contributed to the article and approved the submitted version.

## Conflict of Interest

The authors declare that the research was conducted in the absence of any commercial or financial relationships that could be construed as a potential conflict of interest.

## Publisher's Note

All claims expressed in this article are solely those of the authors and do not necessarily represent those of their affiliated organizations, or those of the publisher, the editors and the reviewers. Any product that may be evaluated in this article, or claim that may be made by its manufacturer, is not guaranteed or endorsed by the publisher.
